# Melanin-like Materials for Photothermal Applications: Recent Advancements and Future Directions

**DOI:** 10.3390/molecules31101712

**Published:** 2026-05-18

**Authors:** Yuan Zou, Jie Deng, Jingluan Yu, Sheng Long, Cheng Chang, Defa Hou, Fulin Yang, Xu Lin

**Affiliations:** 1 National Joint Engineering Research Center for Highly-Efficient Utilization Technology of Forestry Resources, Southwest Forestry University, Kunming 650224, China; yzou24@swfu.edu.cn (Y.Z.);; 2 State Key Laboratory of Advanced Polymer Materials, Sichuan University, Chengdu 610065, China

**Keywords:** melanin, photothermal effect, biomedical, theranostic, environment and energy

## Abstract

Melanin-like polymers, particularly polydopamine, have gained significant attention as photothermal materials due to their broad light absorption (ultraviolet to near-infrared), high photothermal conversion efficiency, negligible fluorescence, good biocompatibility regarding unmodified melanin-like polymers, and universal adhesion. Upon light irradiation, these bioinspired polymers convert absorbed optical energy into heat through molecular vibration and electron–phonon coupling, making them ideal for diverse photothermal applications. This review comprehensively summarizes recent advances in using melanin-like polymers for photothermal purposes. In biomedical engineering, they serve as efficient agents for photothermal therapy and synergistic antibacterial treatment. In catalysis, their photothermal effect enhances pollutant degradation, hydrogen production, and chemical warfare agent detoxification. For water remediation, melanin-like polymers are fabricated into evaporators, membranes, and aerogels for solar-driven steam generation, desalination, and oil spill cleanup. They also enable sensitive photothermal sensing, near-infrared imaging, and laser desorption ionization mass spectrometry imaging. Furthermore, these materials are incorporated into soft actuators and self-healing elastomers for light-controlled shape memory, programmable folding, and remote manipulation. Finally, we discuss remaining challenges such as long-term stability, biocompatibility, scalability, and color limitations and provide future perspectives for advancing melanin-like photothermal materials toward practical applications.

## 1. Introduction

Light is a form of electromagnetic radiation, which can be converted into various types of energy, such as heat, electricity, chemical energy, and photoluminescence [1,2]. Light absorption materials play an important role in the efficient harvesting and utilization of optical energy. Recently, melanin-like polymers have been well established as light-harvesting and photothermal conversion agents due to their broad light absorption spectra and many promising intrinsic properties, such as biocompatibility, universal adhesion, and abundant active groups for surface modification [3,4,5,6,7]. In nature, melanin is a group of biological pigments distributed in a wide range of organisms; it plays an important role in protecting skin from ultraviolet (UV) irradiation and affects the metal ion transport pathway [8,9,10,11,12,13]. In melanocytes, tyrosine can be oxidized enzymatically, forming 3,4-dihydroxyphenylalanine (DOPA), which is able to further self-cyclize, rearrange, and polymerize into eumelanin [14]. Inspired by this synthetic route, numerous melanin-like polymeric materials have been designed and fabricated through the oxidative polymerization of dopamine, DOPA, or 5,6-dihydroxyindole (DHI) [15,16,17]. In particular, polydopamine (PDA) is considered the most typical melanin-like polymer representative; it can be synthesized through the polymerization of dopamine monomers under mild alkaline conditions [18]. The investigation of melanin-like polymers and their derivatives has led to their exponential growth in the biomedical, energy, and environmental fields [19,20,21,22,23,24,25].

Over the past decade, great efforts have been made to develop melanin-like polymers with interesting light absorption and photothermal conversion abilities [26,27,28,29]. They can be processed into various dimensions and morphologies, including fibers [30,31], thin films [32,33], capsules and shells [34,35], nanoparticles (NPs) [4,36,37], and bulk materials (e.g., gels and elastomers) [38,39,40], which all exhibit similar chemical structures to natural melanin, showing broad decay light absorption spectra from the UV to visible and near-infrared regions [3,41]. Therefore, numerous functional materials with light-harvesting and photothermal conversion abilities have been developed and employed in various fields, including biomedical engineering (e.g., photothermal therapy and antibacterial) [35,42,43,44,45,46], catalysts [47], water remediation [48,49,50,51], and sensing and imaging, [52,53], as well as photothermal actuation and self-healing [32,54,55,56].

**Figure 1:**
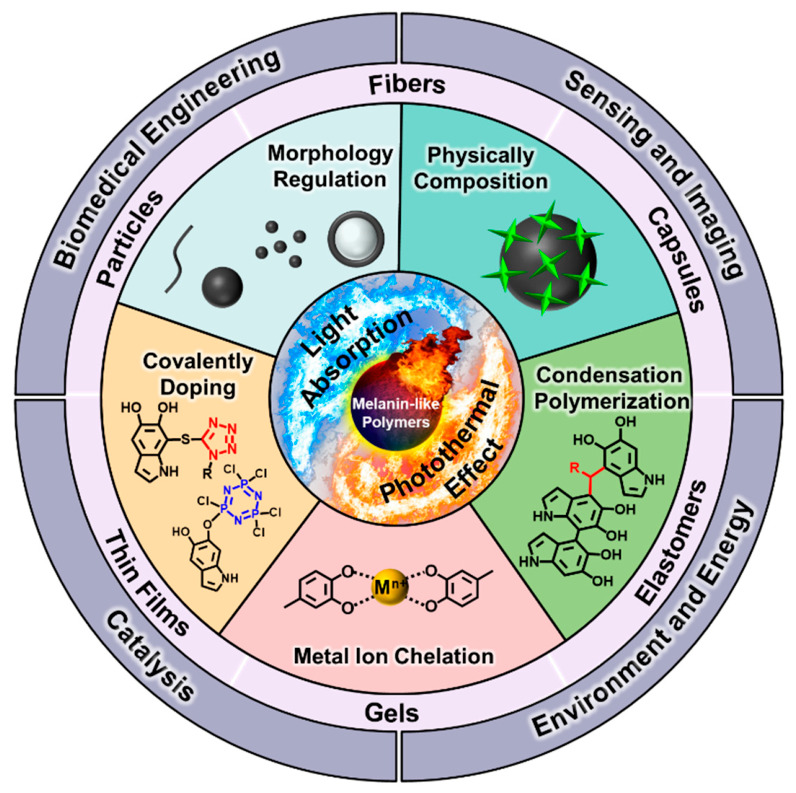
Overview of light absorption and photothermal effect regulation strategies of melanin-like polymers and their applications.

It is known that there is almost no photoluminescence within melanin-like polymers due to their complicated physical stacking and chemical structures, so that regulating their light absorption capacity is critical to the photothermal effects of melanin-like polymers [4,57]. However, the main problem is that the structure–light absorption relationship in melanin-like polymers has not been established systematically so far because of the different oxidation states of the monomers or oligomers and the undefined individual chromophores [4,41]. While some reviews have discussed the regulation of the light absorption and photothermal performance of melanin-like polymers from a methodological perspective [58], the present review focuses on their applications and potential scenarios. In this review, we briefly introduce emerging regulation strategies and then focus on the current progress in melanin-like photothermal functional materials and their potential application scenarios. These melanin-like polymers are divided into five categories: fibers, thin films, capsules and shells, nanoparticles, and bulk materials. These are well documented to be used in a range of applications, such as biomedical engineering, catalysis, water remediation, and sensing and imaging, as well as photothermal actuation and self-healing (Figure 1). The structures and applications of these melanin-like polymers are summarized in Table 1. To guide the selection of melanin-like polymers for specific photothermal applications, Table 2 benchmarks the key parameters of representative materials based on their distinct chemical structures. These parameters include the absorption bandwidth, photothermal conversion efficiency (η_total_), spectral range (NIR-I/II), biocompatibility, and cost-effectiveness. Finally, some remaining challenges and future perspectives are also put forward, aiming to stimulate new inspiration for the development of melanin-like polymers for photothermal applications and beyond.

### Bibliographic Methodology

To ensure transparency and reproducibility, this review followed a systematic literature retrieval process. The databases Web of Science, Scopus, and PubMed were searched using the following keyword combinations: (“melanin-like polymer” OR “polydopamine” OR “PDA” OR “poly(DHI)” OR “poly(DOPA)”) AND (“photothermal” OR “photo-thermal”) AND (“application” OR “therapy” OR “catalysis” OR “water remediation” OR “actuation” OR “self-healing”). The search covered peer-reviewed articles and reviews published between January 2013 and April 2026. Only studies that reported quantitative photothermal performance were considered. Studies focusing solely on non-photothermal properties (e.g., antioxidant, adhesive) were excluded.

Several review articles published in 2025 have addressed specific sub-areas of melanin-based photothermal materials. Wu et al. systematically summarized melanin/melanin-like nanoparticles in tumor photothermal and targeted therapies [129]. Menichetti et al. critically reviewed photothermally controlled release applications of melanin-like nanoparticles [130]. Another recent review focused on polydopamine nanoparticles for antimicrobial applications [131]. The present review is distinguished from these previous works in three key aspects. First, rather than focusing exclusively on nanoparticles, we cover all material morphologies, including fibers, thin films, capsules, gels, and elastomers, thereby providing a more comprehensive material-level perspective. Second, we extend beyond biomedical applications into environmental and energy fields, including solar-driven water remediation, photothermal catalysis, photothermoelectric devices, and soft actuators. Third, we go beyond reporting successful cases by offering critical assessments of material failure scenarios (“rejection criteria”) and a forward-looking roadmap to guide future research. As such, this review complements rather than duplicates existing contributions and serves as a practical reference for researchers across materials science, chemistry, biomedical engineering, and environmental technology.

## 2. Strategies for Regulating Photothermal Performance of Melanin-like Polymers

Before surveying specific photothermal applications, it is instructive to briefly outline the main strategies used to tune the light absorption and photothermal efficiency of melanin-like polymers. These strategies (morphology regulation, hybrid composition, metal ion chelation, covalent doping, and condensation polymerization, as illustrated in Figure 1) are briefly outlined below in terms of their underlying photophysical mechanisms.

(i) Structural monomer modifications: The absorption spectrum of PDA can be shifted by introducing substituents on the monomeric units. For example, ortho-substitution with electron-donating groups or electron-withdrawing groups alters the energy gap between HOMO and LUMO, thereby red- or blue-shifting the absorption [4,10]. Covalent doping with nitrogen-containing heterocycles (e.g., HCCP, CC, TCCA) or mercaptotetrazole (MT) derivatives constructs donor–acceptor pairs that narrow the bandgap and extend absorption into the NIR region [36,37,57,118].
(ii) Metal coordination and LMCT effects: The chelation of transition metal ions (Cu^2+^, Mn^2+^, Fe^3+^, etc.) through catechol groups introduces d-d transitions and ligand-to-metal charge transfer (LMCT), which significantly enhance absorption in the 600–1200 nm range without altering the polymer backbone [17,111,112]. The improved photothermal efficiency directly correlates with metal ion loading and the coordination geometry.
(iii) Control over molecular stacking/aggregation: The broad, decayed absorption of melanin-like polymers is partly due to heterogeneous π-stacking and aggregation. Recent computational studies using time-dependent density functional theory (TD-DFT) and machine learning have revealed how inter-ring dihedral angles (e.g., the twist caused by the carboxylate group in DHICA) affect the conjugation length and absorption profiles [30]. Such tools are now being used to predict optimal monomer sequences and stacking motifs for targeted light absorption.

## 3. Photothermal Applications of Melanin-like Polymers

The excellent light-harvesting properties and photothermal effects of melanin-like polymers mean that they have significant potential for applications in biomedical engineering (e.g., photothermal therapy and antibacterial), catalysis, water remediation, sensing and imaging, photothermal actuation, and self-healing. In recent years, the rational design and effective regulation of their photothermal performance have been well-documented, enabling them to adapt a wide range of practical uses. This section focuses on recent progress regarding advanced applications of melanin-like photothermal materials.

### 3.1. Biomedical Engineering

**Figure 2:**
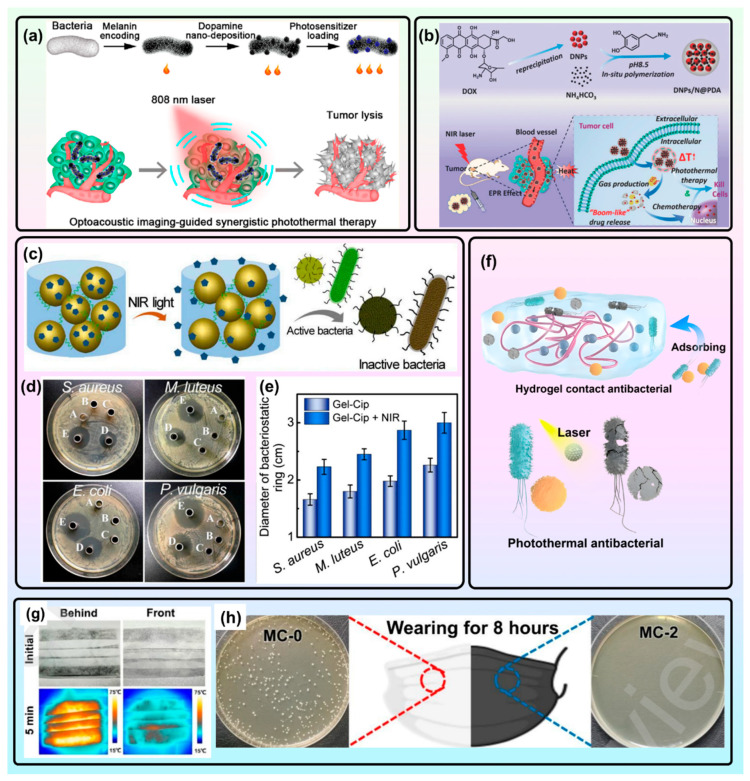
(**a**) Optoacoustic imaging-guided synergistic photothermal therapy through tumor-targeting bacteria coated by PDA. Reproduced with permission [45]. Copyright 2023 American Chemical Society. (**b**) PDA-coated DOX NPs with “bomb-like” on-demand drug release property triggered by NIR light [133]. (**c**) Schematic illustration of Cip-loaded gel and Cip release under NIR light for bacterial inactivation. (**d**) Photographs and (**e**) corresponding statistical histogram of inhibition zones of various bacteria with different treatments (A: NIR, B: Gel, C: Gel + NIR, D: Gel-Cip, and E: Gel-Cip + NIR) after 48 h of culture. (**c**–**e**) Reproduced with permission [120]. Copyright 2019 Elsevier. (**f**) A robust hydrogel (CG/PDA@Ag) with bacterial capture and photothermal antibacterial properties against *E. coli* and *S. aureus* under 808 nm NIR irradiation (1.0 W/cm^2^, 3 min) [135]. (**g**) Infrared photos of the front and rear of a PDA/Fe^3+^/GA-coated surgical mask. (**h**) Results of colony-forming unit assay of blank and PDA/Fe^3+^/GA-coated surgical masks after 8 h of wearing. (**g**,**h**) Reproduced with permission [64]. Copyright 2023 Elsevier.

The intrinsic photothermal effect of PDA-based materials makes them efficient photothermal agents for photothermal therapy [132]. PDA NPs usually exhibit favorable colloidal stability in 10% blood serum solutions, which is beneficial for therapeutic applications in vivo. They also exhibit negligible resonance light scattering, ensuring strong photothermal conversion abilities and excellent photostability, which can help to suppress cancer cell growth without damaging normal cells [3]. In this context, low scattering minimizes light re-emission and energy loss, directing more absorbed energy into nonradiative decay pathways (heat generation). PDA coatings can not only lead to enhanced photothermal effects of metallic nanostructures for photothermal therapy but also suppress their cytotoxicity and improve their dispersity. The PDA capsule can even be coated on tumor-targeting bacteria to achieve biotherapy and photothermal cancer therapy simultaneously (Figure 2a) [35,45]. An all-in-one system with chemo-, gene-, and photothermal treatments can be achieved via a PDA-based nanoplatform for synergistic cancer therapy. For example, MPDA NPs filled with indocyanine green (ICG) and L-arginine (AI-MPDA) have been designed and fabricated, while the photothermal effect of PDA triggers on-demand ICG release to produce oxygen species (ROS) for nitric oxide (NO) gas generation under NIR irradiation [106]. Notably, the loading of ICG and L-arginine cannot weaken the photothermal capabilities of PDA. Hollow PDA nanocapsules possess excellent intrinsic photothermal effects and drug loading abilities for chemo-photothermal synergistic therapy. Through coating DOX NPs with PDA capsules, the drug loading capacity can be as high as 85.8%, resulting in NIR-induced on-demand drug release via the “nanobomb” property through the photothermal effect of PDA (Figure 2b) [133]. Other functional molecules, such as triphenylphosphonium (TPP) and 2-phenylethynesulfonamide (PES), have been immobilized on PDA for bioimaging, chemotherapy, and antibacterial applications, respectively, achieving a synergistic effect with photothermal therapy [74,134].

The excellent photothermal effects of PDA-mediated materials not only induce hyperthermia to destroy the antioxidant stress system of bacteria but also accelerate the generation of antibacterial agents, such as ROS [43,44], NO [77], or antibiotics. For example, PDA–ferrocene (PDA-Fc) coatings have been proven to be redox-active for ROS release to combat bacterial infections [43]. Bacterial biofilm formation can be further hindered under NIR irradiation due to localized hyperthermia and enhanced ROS generation simultaneously. Yu et al. conjugated N-diazeniumdiolate (NONOate) on a PDA capsule through dendritic poly(amidoamine) (PAMAM) for controllable NO release, resulting in synergistic photothermal therapy and NO antibacterial effects [77]. An injectable hydrogel-based drug reservoir was fabricated by blending ciprofloxacin (Cip)-loaded PDA NPs with glycol chitosan, which realized on-demand Cip release for hyperthermia-assisted antimicrobial therapy by NIR light triggering (Figure 2c–e) [120]. The multiple active groups of PDA-based materials are beneficial to capture and kill bacteria through effective interactions, such as hydrophobic interactions, Van der Waals forces, and electrostatic forces (Figure 2f) [135]. A reusable surgical mask with self-sterilization and self-powered real-time respiratory monitoring properties has been developed through the sequential use of PDA and Fe^3+^/gallic acid (GA) coatings. Under sunlight illumination, the mask could achieve self-sterilization, allowing facile reutilization, while real-time respiratory monitoring benefited from the wet electricity generated by the carboxyl groups of GA (Figure 2g,h) [64].

### 3.2. Catalysis

**Figure 3:**
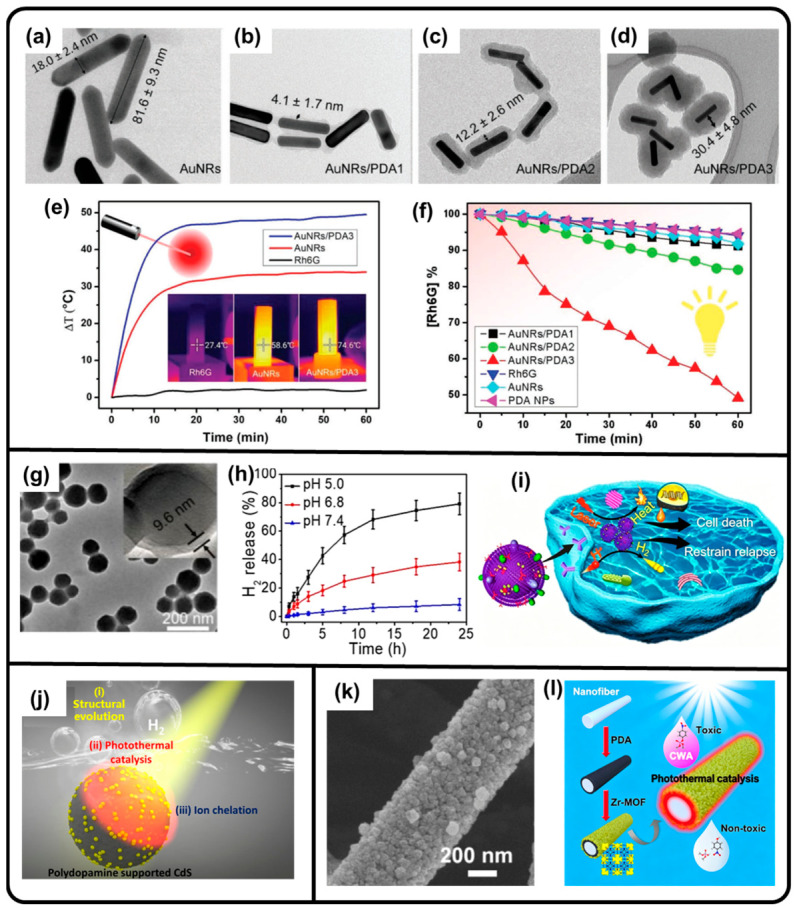
TEM images of (**a**) bare AuNRs and (**b**–**d**) PDA-coated AuNRs with different shell thicknesses. (**e**) Temperature elevation and photographs of Rh6G, bare AuNRs, and PDA-coated AuNRs in solution under NIR irradiation. (**f**) Changes in Rh6G concentration versus time for different photocatalysis applications within 1 h [80]. (**g**) TEM and HRTEM (inset) images of mPDAB NPs. (**h**) The H_2_ release performance of mPDAB under acidic conditions. (**i**) Schematic illustration of anti-inflammation and tumor therapy through photothermal effect and H_2_ generation of mPDAB. (**g**–**i**) Reproduced with permission [137]. Copyright 2019 Elsevier. (**j**) CdS-loaded PDA NPs for photocatalytic H_2_ production through photothermal effect. Reproduced with permission [138]. Copyright 2022 Elsevier. (**k**) SEM image of PDA-mediated Zr-MOF nanofiber. (**l**) PDA-mediated Zr-MOF nanofibers for photothermal-driven CWD degradation. (**k**,**l**) Reproduced with permission [47]. Copyright 2020 American Chemical Society.

PDA can serve as a robust support material for various catalysts. The excellent photothermal effect of PDA can be leveraged to enhance the catalytic activity of photothermal–catalytic reactions. For example, in terms of PDA-coated AuNRs, a PDA coating can enhance the total photothermal effect, leading to the augmentation of ROS production and improving the photocatalytic performance of dye degradation (Figure 3a–f) [80]. The existence of a PDA layer can not only enhance the transference of hot carriers from the AuNR to PDA but also suppress the recombination of the carriers and shuttle them on the particle surface for ROS generation. Moreover, PDA NPs can not only act as photothermal agents but also as carriers of ammonia borane (AB) prodrugs for hydrogen gas (H_2_) production in the weakly acidic conditions of the tumor microenvironment, demonstrating a combination of photothermal and hydrogen therapies for cancer treatment [136,137]. Although this example represents catalytic nanomedicine rather than conventional photothermal-enhanced catalysis, it is included here to illustrate the broader catalytic functionality of melanin-like polymers in biomedical contexts. Due to the intrinsic ability of H_2_ to react with ROS, membrane-camouflaged PDA loaded with AB (mPDAB) could inhibit inflammation during tumor therapy and destroy redox homoeostasis in tumor cells (Figure 3g–i) [137]. Incorporating cadmium sulfide (CdS) onto PDA NPs could also result in stable photothermal activity and photocatalytic behavior for H_2_ production (Figure 3j) [138]. Additionally, other synergistic photocatalytic–photothermal systems can be achieved via PDA-supported catalysts, such as noble metals, bismuth iodide oxide (BiOI), and zirconium-based metal–organic frameworks (Zr-MOFs), for applications such as nitrophenol [115] and p-nitroaniline reduction [116], antibacterial effects [90], and chemical warfare agent (CWA) degradation (Figure 3k,l) [47].

### 3.3. Water Remediation

**Figure 4:**
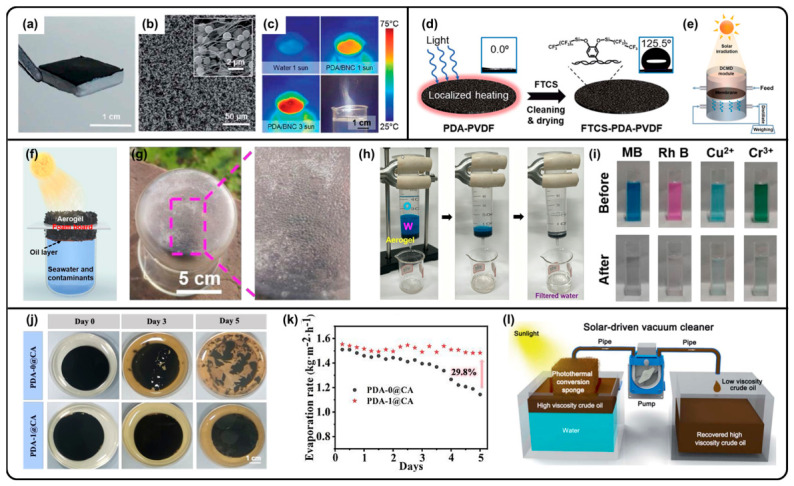
(**a**) Photograph and (**b**) SEM image of PDA-coated BNC foam. (**c**) IR images of water and PDA-coated BNC under 1-sun and 3-sun illumination and photograph of PDA-coated BNC under 3-sun illumination with visible steam generation. (**a**–**c**) Reproduced with permission [48]. Copyright 2017 Royal Society of Chemistry. (**d**) Schematic illustration of synthesis of FTCS-PDA-PVDF membrane and (**e**) solar-driven DCMD system. (**d**,**e**) Reproduced with permission [125]. Copyright 2018 Royal Society of Chemistry. (**f**) Schematic depiction of melanin-like aerogel-based solar desalination device. (**g**) Optical images of outdoor solar steam generation experiment. (**h**) Photographs of oil–water separation and dye removal process. (**i**) Photographs of different contaminant solutions before and after aerogel treatment. (**f**–**i**) Reproduced with permission [124]. Copyright 2023 Royal Society of Chemistry. (**j**) Optical images of PDA- and TOC-PDA-based membrane immersed in bacterial solution for different periods. (**k**) Cycling stability of PDA- and TOC-PDA-based membrane after being immersed in bacterial solution for 5 days. (**j**,**k**) Reproduced with permission [66]. Copyright 2023 Royal Society of Chemistry. (**l**) Schematic illustration of solar-driven vacuum cleaner for continuous separation of high-viscosity crude oil on water surface. Reproduced with permission. Copyright 2018 Royal Society of Chemistry [126].

Solar steam generation is considered a sustainable approach to obtaining fresh water through heat localization by photothermal materials. Melanin-like polymers have been used to fabricate multifunctional photothermal devices, such as gels and thin films [7,48,49,66,124,125,126]. They are robust and universal coatings without selectivity for substrates; they exhibit broad light absorption spectra, high photothermal conversion efficiency, and post-modification properties. For example, a biodegradable photothermal evaporator was fabricated through PDA NP-filled bacterial nanocellulose (BNC), which demonstrated flexible, scalable, and efficient water transportation for solar steam generation (Figure 4a–c) [48]. To resolve the salt deposition problem, fluoro-functional groups (tridecafluoro-1,1,2,2-tetrahydrooctyl)-trichlorosilane (FTCS) can be modified on the surface of a PDA-coated PVDF membrane through the formation of Si-O bonds, resulting in a hydrophobic surface with salt rejection properties in direct contact membrane distillation (DCMD) (Figure 4d,e) [125].

A series of all-in-one melanin-like polymer-based aerogels for water remediation, such as a PDA-coated cellulose aerogel [49] and DHI-formaldehyde aerogel [124], were also developed. They do not only exhibit effective solar-driven evaporation abilities but also absorb contaminants, such as organic dyes, through hydrogen bonding and electrostatic interactions (Figure 4f–i) [49,124]. However, in the real world, sewage is filled with bacteria, which may cause degradation and reduce the photothermal effect of PDA. Notably, including tobramycin (TOB), a natural broad-spectrum antibiotic with a structure of amino-functionalized glycosidic bonds, in the PDA system can achieve the long-term and on-demand release of antibiotics, which could be composed with a cellulose film for durable solar desalination even under bacteria-rich environments (Figure 4j,k) [66].

Furthermore, a hydrophilic PDA-coated melamine sponge could be transformed into an oleophilic sponge through the deposition of polydimethylsiloxane (PDMS), which could reduce the viscosity of crude oil through self-heating for oil spill remediation (Figure 4l) [126].

### 3.4. Sensing and Imaging

**Figure 5:**
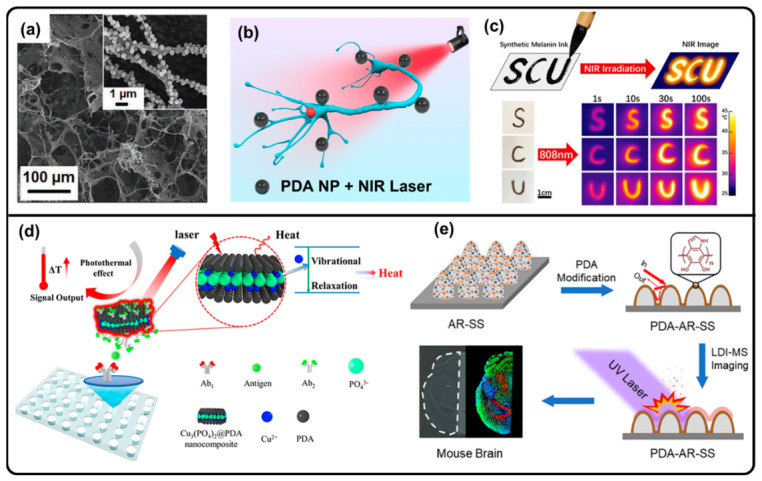
(**a**) SEM image of collagen/PDA NP composite foam (inset: high-magnification SEM image). (**b**) PDA NP-mediated photothermal stimulation of neurons. (**a**,**b**) Reproduced with permission [102]. Copyright 2021 Wiley-VCH. (**c**) Schematic illustration of NIR imaging by PDA inks and IR images of SCU letters under NIR illumination. Reproduced with permission [113]. Copyright 2020 Elsevier. (**d**) Schematic illustration of photothermal immunosensor using PDA-encapsulated Cu3(PO4)2 nanosheets. (**d**) Reproduced with permission [86]. Copyright 2019 American Chemical Society. (**e**) High-sensitivity laser desorption ionization MSI enhanced by photothermal effect of PDA coating through thermal desorption. Reproduced with permission [62]. Copyright 2019 American Chemical Society.

By taking advantage of the excellent photothermal effect, PDA-based materials have been applied for sensing and imaging. PDA NPs have been demonstrated to achieve the reversible modulation of the electrical activity of excitable cells through their photothermal effects, due to their broad light-harvesting abilities, biocompatibility, and biodegradability. A collagen/PDA NP composite foam was fabricated to enhance the interfacing between PDA photothermal transducers and excitable cells, as well as realizing the spatial localization of the photothermal stimulus (Figure 5a,b) [102]. Due to the high blackness and reflective index and good dispersibility in aqueous solution, a series of metal ion-doped PDA NPs with good photothermal behavior as NIR imaging inks were developed (Figure 5c) [113]. PDA-encapsulated Cu_3_(PO_4_)_2_ nanosheets could be adhered to C-reactive protein (CRP) for the photothermal detection and immunosensing of CRP. The temperature changes presented a linear relationship with the CRP concentration from 0.42 to 16 pM, demonstrating a low-cost, biocompatible photothermal immunosensor for protein biomarker detection (Figure 5d) [86]. The light-to-heat conversion of PDA coatings improved the thermal desorption of antireflection polished 304 stainless steel (AR-SS) for high-sensitivity laser desorption ionization mass spectrometry imaging (LDI-MSI) (Figure 5e) [62]. A well-defined contrast agent based on PPy-PDA hybrid-coated silica NPs achieved the amplification of the photoacoustic imaging (PAI) signal intensity, which was dominated by the photothermal effects of the contrast agents [139].

### 3.5. Actuation and Self-Healing

**Figure 6:**
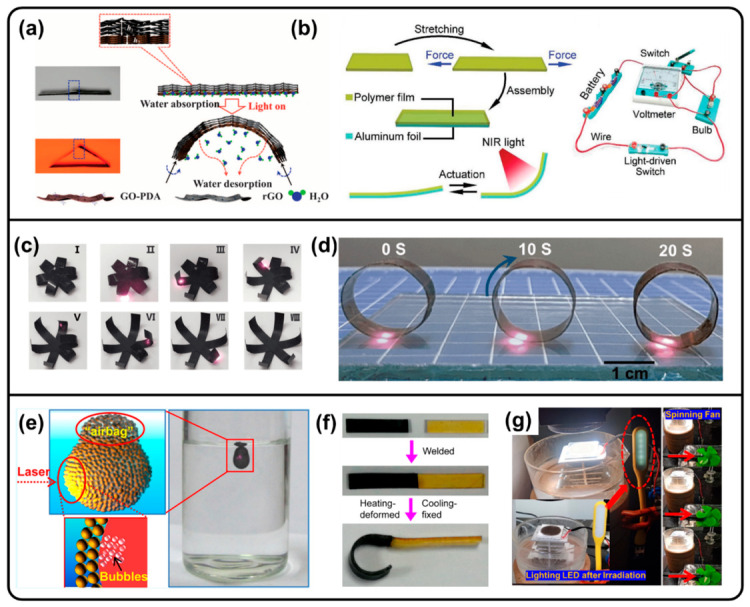
(**a**) Schematic illustration of bending/unbending mechanism of PDA-coated GO/rGO film. Reproduced with permission [32]. Copyright 2015 American Association for the Advancement of Science. (**b**) Schematic illustration of fabrication of bilayer laminate through shape memory polymer and aluminum foil, with light-driven switches applied to control a bulb. Reproduced with permission [140]. Copyright 2021 Wiley-VCH. (**c**) Optical images of shape memory behavior of poly(*L*-DOPA) NP-doped PDMS film triggered by NIR irradiation (I: Closed initial state. II–VII: NIR irradiation sequentially triggers petal-by-petal opening. VIII: Fully recovered, open permanent shape.). (**d**) Optical image of a forward-moving motor driven by NIR irradiation. (**c**,**d**) Reproduced with permission [16]. Copyright 2020 Springer Nature. (**e**) Remote manipulation of a microdroplet encapsulated by core–shell Fe_3_O_4_@PDA NPs by NIR irradiation. Reproduced with permission [73]. Copyright 2016 American Chemical Society. (**f**) Photographs of the self-healing and photothermal deformation performance of PU/PDA composites. Reproduced with permission [127]. Copyright 2022 American Chemical Society. (**g**) Photo-thermoelectric conversion device to light an LED light and turn on a spinning fan. Reproduced with permission [36]. Copyright 2022 American Chemical Society.

PDA coatings with good adhesion and light-to-heat conversion abilities can be used to fabricate light-induced shape transition composites. For example, PDA thin films can not only reduce graphene oxide (GO) to reduced GO (rGO) and provide hydrophilicity for water absorption but also endow the composite with NIR-induced bending/unbending behavior due to the water absorption/desorption abilities, resulting in programmable instant self-folding walking devices (Figure 6a) [32,54]. In addition, PDA-coated shape memory membranes can achieve light-controlled shape programming through the photothermal effect of PDA [55]. Shape memory materials can also be fabricated by PEG-DA and Fe(III) through coordination bonds, with internal stress relaxation properties and broad NIR light absorption abilities. After gluing to aluminum foil, a bilayer actuator could be used as a light-driven switch to achieve the controllable “on–off” switching of a bulb under NIR irradiation (Figure 6b) [140]. Photothermal composite actuators were fabricated through incorporating melanin-like poly(*L*-DOPA) NPs into PDMS films, achieving folding, switching, and forward-moving performance (Figure 6c,d) [16]. Furthermore, PDA-coated Fe_3_O_4_-encapsuled CHCl_3_ droplets could be remotely controlled to ascend, shuttle, and suspend in aqueous solution by NIR laser irradiation due to the generation of small bubbles through the photothermal effect (Figure 6e) [73].

A dynamic crosslinked polyurea/PDA NP nanocomposite has been fabricated with remotely photoresponsive self-healing properties due to the enhanced photothermal effect of PDA NPs (Figure 6f) [127]. The fast self-healing process is facilitated by NIR irradiation, resulting in the dynamic exchange reaction of the hindered urea bonds. Moreover, Diels–Alder (DA) dynamic bonds in crosslinked polyurethane blended with PDA NPs can also enable shape recovery and self-healing through the photothermal effect of PDA, as well as the photothermally dynamic nature of DA bonds [56]. Notably, solar photothermoelectric device can also be fabricated through polyDHI/PES resin composite membranes combined with aluminum plates, which could successfully light an LED light and turn on a spinning fan under sunlight illumination (Figure 6g) [36,37,57,118].

## 4. Discussion

Over the past few years, melanin-like polymers have been well documented as light-harvesting and conversion agents for a wide range of applications, including photothermal therapy, catalysis, water remediation, and soft actuators [3,4,41]. Despite these successes, several critical challenges remain that hinder their further development and practical translation.

Firstly, the intrinsic dark color of most melanin-like polymers limits their application in cosmetics, sunscreens, and transparent devices. While broadening the bandgap can reduce visible and NIR absorption while enhancing UV shielding, achieving light-colored or transparent melanin-like materials remains a significant hurdle. Future efforts should focus on molecular engineering to disrupt long-range π-conjugation without sacrificing UV protection, e.g., by introducing twisted monomer units such as DHICA or aliphatic spacers.

Secondly, although melanin-like polymers are generally considered biocompatible due to their natural origin, strategies to enhance light absorption often rely on introducing non-natural heterocyclic compounds or high concentrations of metal ions. The long-term biological safety of such modified materials requires systematic evaluation. Moreover, increasing the quinone content, a common approach to boost absorption, may compromise the intrinsic adhesion and solubility of polydopamine and its analogs [15,16]. To address these issues, we suggest a shift toward naturally derived building blocks (e.g., gallic acid, caffeic acid) and tighter control over metal ion loading to avoid excess leaching, combined with long-term in vivo degradation studies.

Thirdly, the polymerization mechanism of melanin-like polymers remains incompletely understood. The precise chemical structures, the identity and distribution of chromophoric units (e.g., DHI, DHICA, indolequinone), and the polymerization pathways under different conditions (acidic, alkaline, radical-mediated) are still debated. A clearer structure–property relationship is essential for rational material design. Computational simulations and machine learning approaches have recently been applied to predict absorption spectra and molecular conformations, offering promising tools to unravel these complexities. Specifically, for extending absorption into the NIR-II window (1000–1700 nm), machine learning could guide the design of donor–acceptor pairs or quinoid-rich domains, while synthetic strategies such as condensation polymerization or covalent doping with strong acceptors should be systematically explored.

Fourthly, the gap between laboratory-scale investigations and real-world applications is substantial. Photostability is a major concern: polyphenolic moieties are prone to oxidation under prolonged light exposure, leading to the gradual loss of photothermal performance. Additionally, small oligomers or unreacted monomers may leach out from the polymer network, especially in aqueous environments, raising concerns about environmental and biological safety. Furthermore, the synthetic yield of artificial melanin is typically very low, making large-scale production cost-prohibitive. To overcome these barriers, encapsulation strategies such as using silica or polymer shells could be employed to slow photooxidation, while covalent crosslinking may help to reduce monomer or oligomer leaching. Furthermore, developing continuous flow or enzyme-catalyzed polymerization methods would improve synthetic yields and scalability. These limitations have different weight depending on the application: for water remediation membranes (Section 3.3), leaching and oxidative stability are the primary concerns; for therapeutic applications (Section 3.1), biodegradation kinetics and the clearance of metabolites are most critical; for catalysis (Section 3.2), photostability under prolonged irradiation dominates.

Beyond visible and NIR absorption, other types of melanin—such as pheomelanin and allomelanin (e.g., fungal melanin)—have been shown to absorb shorter-wavelength radiation, including UV, gamma, and X-rays. Fungal melanin, found in high-radiation environments like Chernobyl, is particularly promising as a radioprotective material [141,142]. Expanding the scope of melanin-like polymers to cover the entire electromagnetic spectrum could open up new avenues in radiation shielding and space applications.

We also acknowledge the limited understanding of material failure under realistic conditions. First, the long-term photostability of melanin-like polymers remains a concern; prolonged light exposure gradually reduces their photothermal efficiency due to photooxidation [4]. Second, metal ions chelated for enhanced NIR absorption may leach out under acidic or physiologically relevant conditions, and unreacted monomers or oligomers can also diffuse from the polymer network, raising safety concerns for in vivo applications [27,29]. Third, the biodegradation kinetics of these materials in complex biological environments (e.g., serum, tissue, or microbial communities) are poorly characterized [64,66]. While PDA degrades slowly over weeks to months, the actual degradation rate varies significantly with the local pH, enzyme activity, and protein adsorption. These factors must be carefully considered when designing melanin-like polymers for long-term implants or rapidly clearing devices.

Lastly, while the melanin-like polymer family also includes poly(norepinephrine), synthetic fungal melanin, and other polyphenolic platforms, the present review focuses on those with well-established photothermal performance, i.e., polydopamine (PDA), poly(DHI), and their derivatives [19,142,143]. Compared to gold nanoparticles and MXenes, which exhibit narrow plasmonic absorption peaks, melanin-like polymers offer broad absorption from UV to NIR without shape engineering. Relative to carbon-based nanomaterials (carbon nanotubes, graphene oxide), melanin-like polymers are intrinsically biocompatible, biodegradable, and rich in surface-functional groups for post-modification, although their photothermal conversion efficiency per mass is generally lower. Hence, melanin-like polymers are best suited for applications where moderate yet broadband absorption, safety, and chemical versatility are prioritized, such as in injectable hydrogels, biodegradable coatings, and theranostic platforms.

## 5. Conclusions

In conclusion, melanin-like polymers have proven themselves as highly versatile and efficient photothermal materials, finding promising applications in cancer therapy, antibacterial treatment, catalysis, water remediation, sensing, imaging, actuation, and self-healing. Despite this remarkable progress, several critical challenges remain. The intrinsic dark color limits their use in cosmetics and transparent devices, although melanin’s natural dark hue can actually function as a constituent for generating structural colors through orderly arrangement at the nanoscale (e.g., feather barbules) [144,145]. Moving from color to performance, enhancing light absorption often introduces non-natural components that may compromise biocompatibility. Moreover, the polymerization mechanisms and structure–property relationships are still not fully understood, and practical issues such as poor photostability, oligomer leaching, and low synthetic yields hinder large-scale production. Overcoming these obstacles will require close collaboration across polymer chemistry, materials engineering, and computational modeling. Nevertheless, with continued innovation in synthesis and material design, melanin-like polymers hold great promise to become key components in next-generation photothermal technologies for sustainable energy, clean water, precision medicine, and soft robotics.

## Figures and Tables

**Table 1:** Melanin-like polymeric photothermal materials for various applications.

Category	Photothermal Material	Application	Ref.
Fibers	PDA-mediated Zr-MOF fiber	Photothermal catalysis	[47]
	PPC@PDA nanofiber	Photothermal therapy, would healing	[59]
	PDA/hydroxyapatite NW	Water remediation	[60]
	PDA/PEI/PPy@PI nanofiber	Solar steam generation	[61]
	PDA/alginate nanofiber	Solar steam generation, solar electric power	[30]
	AgNW@PDA	Photothermal therapy	[44]
Thin films	PDA coating	Laser desorption ionization MSI	[62]
		Photothermal antibacterial	[63]
	PDA/GA/Fe^3+^ coating	Self-sterilization and self-powered real-time respiratory monitoring	[64]
	PDA-Fc coating	Photothermal antibacterial	[43]
	PDA-APN coating	Antibacterial, osteogenesis	[65]
	Nafion/PDA composite film	Shape memory	[55]
	PDA-RGO layer	Photothermal actuator	[32,54]
	TOB-doped PDA coating	Antibacterial, water remediation	[66]
	LAPDA coating	Scratch resistance, biofouling resistance	[33]
Capsules and shells	Hollow PDA NP	Photothermal therapy	[67]
		Photothermal immunotherapy	[68]
	MSN@PDA NP	Chemo/gene/photothermal therapy	[69]
		Photothermal immunotherapy	[70]
	MSN@PDA-HA NP	Chemo-photothermal therapy	[71]
	BGN@PDA NP	Photothermal therapy, bone regeneration	[72]
	Fe_3_O_4_@PDA NP	Remote manipulation of a microdroplet	[73]
		Photothermal antibacterial	[74]
		Photothermal therapy	[75,76]
	Fe_3_O_4_@PDA@PAMAM@NONOate	Photothermal and NO antibacterial	[77]
	Au@PDA nanoworm	Imaging, delivery, photothermal therapy	[78]
	AuNR@PDA NP	Drug delivery, photothermal therapy	[79]
		Photocatalysis	[80]
	AuNS@PDA NP	Chemo-photothermal therapy	[81]
	RGD-125IPt-AuNR@PDA NP	Chemo-photothermal therapy	[82]
	Cu(II)-AuNR@PDA NP	Theranostics	[83]
	AuNS@PDA NP	Photothermal therapy	[84]
	MnS@PDA NP	MRI, photothermal therapy	[85]
	MoS_2_@PDA NP	Photothermal antibacterial	[44]
	Cu_3_(PO_4_)_2_@PDA NP	Photothermal detection of immunosensing	[86]
	Bi_2_Se_3_@PDA NP	Drug delivery, photothermal therapy	[87]
	Mesoporous CoFe_2_O_4_@PDA NP	Drug delivery, photothermal therapy	[88]
	CoP@PDA NP	MRI, chemo-photothermal therapy	[89]
	BiO_1_-xI@PDA NP	Diagnosis, photothermal therapy	[90]
	TiO_2_-x@PDA NP	Chem/photodynamic/photothermal Therapy	[91]
	Mn_3_O_4_@PDA NP	Chemo-photothermal therapy	[92]
	DOX-HCF@PDA NP	Chemo-photothermal therapy	[93]
	MOF@PDA NP	Photothermal therapy	[94]
		Chemo-thermotherapy	[95]
	UCNPs@PDA-ICG	Photothermal/photodynamic therapy	[96]
	PLGA@PDA NP	Chemo-photothermal therapy	[97]
	PS@PDA NP	Mucopenetration	[98]
	PDA-coated amphiphilic copolymer NP	Chemo-/gene/photothermal therapy	[99]
	DOX@PDA–bortezomib NP	Chemo-photothermal therapy	[100]
	PDA-coated nucleic acid NP	Photothermal therapy	[101]
	PDA-coated bacteria	Photothermal therapy	[35,45]
	Gd-TMV@PDA NP	MRI, photothermal therapy	[53]
NPs	PDA NP	Modulation of electrical activity of cells	[102]
		Photothermal therapy	[3]
	TEMPO-doped PDA NP	Solar steam generation	[4]
	Arginine-doped PDA NP	Photothermal therapy	[42]
	TCCA-doped PDA NP	Photothermoelectricity, Marangoni actuator	[36]
	HCCP-doped PDA NP	Photothermoelectricity	[37]
	CC-doped PDA NP	Photothermoelectricity	[37]
	MT-doped PDA NP	Photothermoelectricity, solar steam generation	[57]
	PDA@MXene NP	Solar steam generation	[103]
	DOX-EGCG/PDA-FA NP	Chemo-photothermal therapy	[104]
	PDA@CP-PEG-DOX NP	Chemo-photothermal combination therapy	[105]
	AI-MPDA NP	Photodynamic and photothermal therapy	[106]
	CaP-MPDA NP	Photothermal and siRNA therapy	[107]
	MSA-2-loaded MPDA@Mn NP	Photothermal-immunotherapy	[108]
	Fe-MPDA@Sorafenib–triphenylphosphine NP	Ferroptosis, photothermal therapy	[109]
	MPDA@ bacterial membrane vesicles	Photothermal therapy	[110]
	Cu(II)-PDA NP	MRI, chemo-photothermal therapy	[111]
	Mn(II)-PDA NP	MRI, photothermal therapy	[112]
	Metal ion-doped PDA NP	NIR imaging inks	[113]
	Fe(II)-PDA@PEG-RCD NP	Ferroptosis	[114]
	Pd-PDA NP	Photothermal catalysis	[115]
	Au@PDA-SiO_2_-PNIPAm NP	Photothermal catalysis	[116]
	Poly(DOPA) NP	Photothermal therapy	[117]
	Metal ion-doped Poly(DOPA) NP	Photothermal actuation	[16]
	Poly(DHI) NP	Photothermoelectricity	[118]
Gels	PDA NPs/PNIPAM hydrogel	Drug delivery, self-healing	[119]
	PDA NP-knotted glycol chitosan hydrogel	Drug delivery, antibacterial	[120]
	PDA NP-knotted PEG hydrogel	Drug delivery, chemo-photothermal therapy	[38]
	PDA/BNC hydrogel	Solar steam generation	[48]
	PDA/gelatin hydrogel	Drug delivery, photothermal therapy	[39]
	Alg-PDA hydrogel	Photothermal therapy, tissue repair	[121]
	AuNP@PDA-PNAm composite hydrogel	Theranostic, mammoplastic	[122]
	Double network hydrogel	Antibacterial, wood dressing	[123]
	Poly(DHI) aerogel	Water remediation	[124]
	PDA-filled cellulose aerogel	Water remediation	[49]
	PDA/BNC aerogel	Water remediation	[125]
	PDMS/PDA-coated melamine sponge	Crude oil spill remediation	[126]
Elastomers	PDA NP-doped PU elastomer	Self-healing, photoactuation	[56,127]
	PDA NP-doped liquid crystal elastomer	Photothermal actuation	[128]

**Table 2:** Benchmarking of representative melanin-like polymers for photothermal applications.

Material	Absorption Bandwidth (nm)	Excitation λ (nm)	Power Density (W/cm^2^)	Conc. (mg/mL)	ΔT (°C)	η_total_ (%)	Spectral Range (NIR-I/II)	Biocompatibility	Cost-Effectiveness	Ref.
PDA	200–800 (broad, decay)	808	2	0.1	23–25	13.1–16.7	NIR-I (weak)	Excellent	Medium	[4]
Poly(DHI)	300–900 (more uniform)	808	2	0.1	37–57	18.5–27.8	NIR-I (moderate)	Excellent	High	[118]
Poly(L-DOPA)	200–900	808	2	0.1	~51	17.6–27.4	NIR-I (moderate)	Excellent	Medium	[16]
TEMPO-doped PDA	300–1100	808	2	0.1	30–48	18.6–29.1	NIR-I and II	Good	Medium	[4]
MT-doped PDA	350–1200	808	2	0.1	36–45	23.5–28.3	NIR-I and II	Good	Medium	[57]
Arginine-doped PDA	600–1200	808	1.5	0.1	12–20	26.4–27.5	NIR-I and II	Good	Medium	[42]

## Data Availability

The data contained within this review are available by accessing the articles cited in the bibliography. No new data were created for this review.

## Abbreviations

PDA	polydopamine
NP	nanoparticle
MPDA	mesoporous polydopamine
DHI	5,6-dihydroxyindole
DHICA	5,6-dihydroxyindole-2-carboxylic acid
DOPA	3,4-dihydroxyphenylalanine
NIR	near-infrared
UV	ultraviolet
ROS	reactive oxygen species
BNC	bacterial nanocellulose
rGO	reduced graphene oxide
LMCT	ligand-to-metal charge transfer
ICG	indocyanine green
PAMAM	poly(amidoamine)
NONOate	N-diazeniumdiolate
TCCA	trichloroisocyanuric acid
HCCP	hexachlorocyclotriphosphazene
CC	cyanogen chloride
MT	mercaptotetrazole (and derivatives MMT, PMT, NMT)
APN	adiponectin
TOB	tobramycin
LAPDA	laser-annealed polydopamine
MSN	mesoporous silica nanoparticle
BGN	bioactive glass nanoparticle
UCNPs	upconversion nanoparticles
TEMPO	2,2,6,6-tetramethylpiperidinyl-1-oxide
EGCG	epigallocatechin gallate
RGD	arginine–glycine–aspartic acid peptide

## References

[1] Ruggenthaler M., Tancogne-Dejean N., Flick J., Appel H., Rubio A. (2018). From a quantum-electrodynamical light–matter description to novel spectroscopies. Nat. Rev. Chem..

[2] Aydin K., Ferry V.E., Briggs R.M., Atwater H.A. (2011). Broadband polarization-independent resonant light absorption using ultrathin plasmonic super absorbers. Nat. Commun..

[3] Liu Y., Ai K., Liu J., Deng M., He Y., Lu L. (2013). Dopamine-Melanin Colloidal Nanospheres: An Efficient Near-Infrared Photothermal Therapeutic Agent for In Vivo Cancer Therapy. Adv. Mater..

[4] Zou Y., Chen X., Yang P., Liang G., Yang Y., Gu Z., Li Y. (2020). Regulating the absorption spectrum of polydopamine. Sci. Adv..

[5] Cao H., Yang L., Tian R., Wu H., Gu Z., Li Y. (2022). Versatile polyphenolic platforms in regulating cell biology. Chem. Soc. Rev..

[6] Zhang T., Hu J., Guo L., Gu Z., Jiang X., Li Y. (2024). Nature-inspired safe and efficient hair dyes: Beyond the traditional hair dyes. Green Chem..

[7] Li M., Bai W., Yang Y., Zhang X., Wu H., Li Y., Xu Y. (2024). Waste Tea-Derived Theabrownins for Solar-Driven Steam Generation. ACS Appl. Mater. Interfaces.

[8] d’Ischia M., Wakamatsu K., Cicoira F., Di Mauro E., Garcia-Borron J.C., Commo S., Galván I., Ghanem G., Kenzo K., Meredith P. (2015). Melanins and melanogenesis: From pigment cells to human health and technological applications. Pigment Cell Melanoma Res..

[9] Hong L., Simon J.D. (2007). Current Understanding of the Binding Sites, Capacity, Affinity, and Biological Significance of Metals in Melanin. J. Phys. Chem. B.

[10] Yang Z., Wang X., Bai W., Zou Z., Wu H., Li Y. (2024). Structural disruption of melanin-like polymers with boosted UV protection. Sci. China Chem..

[11] Cao H., Zhu J., Zhang J., Yang L., Guo X., Tian R., Wu H., Li Y., Gu Z. (2023). In Situ Fabrication of Robust Polyphenolic Hydrogels for Skin Protection and Repair. Chem. Mater..

[12] Wang T., Zhao J., Yang Z., Xiong L., Li L., Gu Z., Li Y. (2022). Polyphenolic sunscreens for photoprotection. Green Chem..

[13] Hu J., Yang L., Yang P., Jiang S., Liu X., Li Y. (2020). Polydopamine free radical scavengers. Biomater. Sci..

[14] Simon J.D., Peles D.N. (2010). The Red and the Black. Acc. Chem. Res..

[15] Liu Y., Ai K., Lu L. (2014). Polydopamine and Its Derivative Materials: Synthesis and Promising Applications in Energy, Environmental, and Biomedical Fields. Chem. Rev..

[16] Wang X., Yang L., Yang P., Guo W., Zhang Q.-P., Liu X., Li Y. (2020). Metal ion-promoted fabrication of melanin-like poly(L-DOPA) nanoparticles for photothermal actuation. Sci. China Chem..

[17] Chang C., Long S., Deng J., Shen X., Sun H., Lin X., Zou Y. (2026). Engineering the Melanin Building Block DHICA into a Fe3+ Coordination Coating for Solar Evaporation. Langmuir.

[18] Lee H., Dellatore S.M., Miller W.M., Messersmith P.B. (2007). Mussel-Inspired Surface Chemistry for Multifunctional Coatings. Science.

[19] Zhang H., Huang C., Zhang J., Wang C., Wang T., Shi S., Gu Z., Li Y. (2022). Synthetic fungal melanin nanoparticles with excellent antioxidative property. Giant.

[20] Yang P., Zhu F., Zhang Z., Cheng Y., Wang Z., Li Y. (2021). Stimuli-responsive polydopamine-based smart materials. Chem. Soc. Rev..

[21] Yang L., Guo X., Jin Z., Guo W., Duan G., Liu X., Li Y. (2021). Emergence of melanin-inspired supercapacitors. Nano Today.

[22] Jin Z., Yang L., Shi S., Wang T., Duan G., Liu X., Li Y. (2021). Flexible Polydopamine Bioelectronics. Adv. Funct. Mater..

[23] Fu Y., Yang L., Zhang J., Hu J., Duan G., Liu X., Li Y., Gu Z. (2021). Polydopamine antibacterial materials. Mater. Horiz..

[24] Koshevaya E., Lifanovsky N., Shishmakova E., Staltsov M., Dubovik A., Belousov A., Kaluzhny D., Kuzmin V., Morozov V., Kolyvanova M. (2026). Interaction of BSA with Ta_2_O_5_ Nanoparticles: The Effect of Polydopamine Pre-Coating. Molecules.

[25] Lo Presti A., Molinari F.N., Abate C., Fazio E., Corsaro C., Giuffrè O., Piperno A., Neri G., Foti C. (2026). Electrochemical Detection of Levofloxacin Using a Polydopamine-Based Molecular Imprinting Polymer. Molecules.

[26] Farokhi M., Mottaghitalab F., Saeb M.R., Thomas S. (2019). Functionalized theranostic nanocarriers with bio-inspired polydopamine for tumor imaging and chemo-photothermal therapy. J. Control. Release.

[27] Wang Z., Zou Y., Li Y., Cheng Y. (2020). Metal-Containing Polydopamine Nanomaterials: Catalysis, Energy, and Theranostics. Small.

[28] Cheng W., Zeng X., Chen H., Li Z., Zeng W., Mei L., Zhao Y. (2019). Versatile Polydopamine Platforms: Synthesis and Promising Applications for Surface Modification and Advanced Nanomedicine. ACS Nano.

[29] Li N., Zou Y., Zhang X., Jin Z., Yang Y., Yang L., Duan G., Xu Y., Li Y. (2022). Degradable and Recyclable Solar Desalination Membranes Based on Naturally Occurring Building Blocks. Chem. Mater..

[30] Zong L., Li M., Li C. (2018). Intensifying solar-thermal harvest of low-dimension biologic nanostructures for electric power and solar desalination. Nano Energy.

[31] Ding W., Chechetka S.A., Masuda M., Shimizu T., Aoyagi M., Minamikawa H., Miyako E. (2016). Lipid Nanotube Tailored Fabrication of Uniquely Shaped Polydopamine Nanofibers as Photothermal Converters. Chem. Eur. J..

[32] Mu J., Hou C., Wang H., Li Y., Zhang Q., Zhu M. (2015). Origami-inspired active graphene-based paper for programmable instant self-folding walking devices. Sci. Adv..

[33] Lee K., Park M., Malollari K.G., Shin J., Winkler S.M., Zheng Y., Park J.H., Grigoropoulos C.P., Messersmith P.B. (2020). Laser-induced graphitization of polydopamine leads to enhanced mechanical performance while preserving multifunctionality. Nat. Commun..

[34] Hu C., Chen H., Zheng J., Zhou S., Yang X., Ngocho K., Xie T., Wang K., Liu J. (2024). Interfacial Polymerization Fabricated Polydopamine Capsules as a Step Toward a Photothermal Protocell Model. Adv. Funct. Mater..

[35] Chen W., Wang Y., Qin M., Zhang X., Zhang Z., Sun X., Gu Z. (2018). Bacteria-Driven Hypoxia Targeting for Combined Biotherapy and Photothermal Therapy. ACS Nano.

[36] Bai W., Xiang P., Liu H., Guo H., Tang Z., Yang P., Zou Y., Yang Y., Gu Z., Li Y. (2022). Molecular Hyperpolarization-Directed Photothermally Enhanced Melanin-Inspired Polymers. Macromolecules.

[37] Bai W., Yang P., Liu H., Zou Y., Wang X., Yang Y., Gu Z., Li Y. (2022). Boosting the Optical Absorption of Melanin-like Polymers. Macromolecules.

[38] Wang X., Wang C., Wang X., Wang Y., Zhang Q., Cheng Y. (2017). A Polydopamine Nanoparticle-Knotted Poly(ethylene glycol) Hydrogel for On-Demand Drug Delivery and Chemo-photothermal Therapy. Chem. Mater..

[39] He G., Chen S., Xu Y., Miao Z., Ma Y., Qian H., Lu Y., Zha Z. (2019). Charge reversal induced colloidal hydrogel acts as a multi-stimuli responsive drug delivery platform for synergistic cancer therapy. Mater. Horiz..

[40] Fu Y., Wan R., Yang L., Xiong L., Hu J., Tang J., He H., Gu Z., Li L., Li Y. (2022). Propolis inspired sunscreens for efficient UV-protection and skin barrier maintenance. Nano Res..

[41] Hong S., Wang Y., Park S.Y., Lee H. (2018). Progressive fuzzy cation-π assembly of biological catecholamines. Sci. Adv..

[42] Yang P., Zhang S., Zhang N., Wang Y., Zhong J., Sun X., Qi Y., Chen X., Li Z., Li Y. (2019). Tailoring Synthetic Melanin Nanoparticles for Enhanced Photothermal Therapy. ACS Appl. Mater. Interfaces.

[43] Song J., Liu H., Lei M., Tan H., Chen Z., Antoshin A., Payne G.F., Qu X., Liu C. (2020). Redox-Channeling Polydopamine-Ferrocene (PDA-Fc) Coating to Confer Context-Dependent and Photothermal Antimicrobial Activities. ACS Appl. Mater. Interfaces.

[44] Yuan Z., Tao B., He Y., Liu J., Lin C., Shen X., Ding Y., Yu Y., Mu C., Liu P. (2019). Biocompatible MoS_2_/PDA-RGD coating on titanium implant with antibacterial property via intrinsic ROS-independent oxidative stress and NIR irradiation. Biomaterials.

[45] Guo H., Cao Z., Li J., Fu Z., Lin S., Wang L., Liu J. (2023). Integrating Bacteria with a Ternary Combination of Photosensitizers for Monochromatic Irradiation-Mediated Photoacoustic Imaging-Guided Synergistic Photothermal Therapy. ACS Nano.

[46] Chen M.-X., Dai J.-Y., Zhang L.-Y., Wang S.-P., Liu J.-K., Wu Y.-G., Ba X.-W., Liu X.-Q. (2024). The Role of Renewable Protocatechol Acid in Epoxy Coating Modification: Significantly Improved Antibacterial and Adhesive Properties. Chin. J. Polym. Sci..

[47] Yao A., Jiao X., Chen D., Li C. (2020). Bio-Inspired Polydopamine-Mediated Zr-MOF Fabrics for Solar Photothermal-Driven Instantaneous Detoxification of Chemical Warfare Agent Simulants. ACS Appl. Mater. Interfaces.

[48] Jiang Q., Gholami Derami H., Ghim D., Cao S., Jun Y.-S., Singamaneni S. (2017). Polydopamine-filled bacterial nanocellulose as a biodegradable interfacial photothermal evaporator for highly efficient solar steam generation. J. Mater. Chem. A.

[49] Zou Y., Zhao J., Zhu J., Guo X., Chen P., Duan G., Liu X., Li Y. (2021). A Mussel-Inspired Polydopamine-Filled Cellulose Aerogel for Solar-Enabled Water Remediation. ACS Appl. Mater. Interfaces.

[50] Zhang X., Yan Y., Li N., Yang P., Yang Y., Duan G., Wang X., Xu Y., Li Y. (2023). A robust and 3D-printed solar evaporator based on naturally occurring molecules. Sci. Bull..

[51] Xu Y., Hu J., Zhang X., Yuan D., Duan G., Li Y. (2022). Robust and multifunctional natural polyphenolic composites for water remediation. Mater. Horiz..

[52] Lemaster J.E., Wang Z., Hariri A., Chen F., Hu Z., Huang Y., Barback C.V., Cochran R., Gianneschi N.C., Jokerst J.V. (2019). Gadolinium Doping Enhances the Photoacoustic Signal of Synthetic Melanin Nanoparticles: A Dual Modality Contrast Agent for Stem Cell Imaging. Chem. Mater..

[53] Hu H., Yang Q., Baroni S., Yang H., Aime S., Steinmetz N.F. (2019). Polydopamine-decorated tobacco mosaic virus for photoacoustic/magnetic resonance bimodal imaging and photothermal cancer therapy. Nanoscale.

[54] Ji M., Jiang N., Chang J., Sun J. (2014). Near-Infrared Light-Driven, Highly Efficient Bilayer Actuators Based on Polydopamine-Modified Reduced Graphene Oxide. Adv. Funct. Mater..

[55] Chen T., Li H., Li Z., Jin Q., Ji J. (2016). A “writing” strategy for shape transition with infinitely adjustable shaping sequences and in situ tunable 3D structures. Mater. Horiz..

[56] Yang L., Lu X., Wang Z., Xia H. (2018). Diels–Alder dynamic crosslinked polyurethane/polydopamine composites with NIR triggered self-healing function. Polym. Chem..

[57] Zou Y., Liu H., Wang X., Xu Y., Yang Y., Bai W., Li Y. (2023). Light Harvesting Modulation of Melanin-Like Polymers via Thiol-Michael “Click” Chemistry. Macromolecules.

[58] Zou Y., Wang T., Lin X., Yang L., Li Y. (2025). Regulation of the Light Absorption and Photothermal Performance of Melanin-Like Polymers. Acc. Chem. Res..

[59] Xi Y., Ge J., Wang M., Chen M., Niu W., Cheng W., Xue Y., Lin C., Lei B. (2020). Bioactive Anti-inflammatory, Antibacterial, Antioxidative Silicon-Based Nanofibrous Dressing Enables Cutaneous Tumor Photothermo-Chemo Therapy and Infection-Induced Wound Healing. ACS Nano.

[60] Cao S., Wu X., Zhu Y., Gupta R., Tan A., Wang Z., Jun Y.-S., Singamaneni S. (2020). Polydopamine/hydroxyapatite nanowire-based bilayered membrane for photothermal-driven membrane distillation. J. Mater. Chem. A.

[61] Xu Y., Xu H., Zhu Z., Hou H., Zuo J., Cui F., Liu D., Wang W. (2019). A mechanically durable, sustained corrosion-resistant photothermal nanofiber membrane for highly efficient solar distillation. J. Mater. Chem. A.

[62] Yang J., Zhang W., Zhang H., Zhong M., Cao W., Li Z., Huang X., Nie Z., Liu J., Li P. (2019). Polydopamine-Modified Substrates for High-Sensitivity Laser Desorption Ionization Mass Spectrometry Imaging. ACS Appl. Mater. Interfaces.

[63] Lei W., Ren K., Chen T., Chen X., Li B., Chang H., Ji J. (2016). Polydopamine Nanocoating for Effective Photothermal Killing of Bacteria and Fungus upon Near-Infrared Irradiation. Adv. Mater. Interfaces.

[64] Li H., Li N., Yang Y., Zhang L., Bai W., Zhang X., Xu Y., Li Y. (2023). Self-sterilization and self-powered real-time respiratory monitoring of reusable masks engineered by bioinspired coatings. Nano Energy.

[65] Deng Y., Gao X., Shi X.-L., Lu S., Yang W., Duan C., Chen Z.-G. (2020). Graphene Oxide and Adiponectin-Functionalized Sulfonated Poly(etheretherketone) with Effective Osteogenicity and Remotely Repeatable Photodisinfection. Chem. Mater..

[66] Yang Y., Yang L., Yang F., Bai W., Zhang X., Li H., Duan G., Xu Y., Li Y. (2023). A bioinspired antibacterial and photothermal membrane for stable and durable clean water remediation. Mater. Horiz..

[67] Tan L., Tang W., Liu T., Ren X., Fu C., Liu B., Ren J., Meng X. (2016). Biocompatible Hollow Polydopamine Nanoparticles Loaded Ionic Liquid Enhanced Tumor Microwave Thermal Ablation in Vivo. ACS Appl. Mater. Interfaces.

[68] Guo K., Chen D., Ren S., Younis M.R., Teng Z., Zhang L., Wang Z., Tian Y. (2023). Reversing Immune Suppression and Potentiating Photothermal Immunotherapy via Bispecific Immune Checkpoint Inhibitor Loaded Hollow Polydopamine Nanospheres. ACS Appl. Mater. Interfaces.

[69] Cheng W., Nie J., Gao N., Liu G., Tao W., Xiao X., Jiang L., Liu Z., Zeng X., Mei L. (2017). A Multifunctional Nanoplatform against Multidrug Resistant Cancer: Merging the Best of Targeted Chemo/Gene/Photothermal Therapy. Adv. Funct. Mater..

[70] Huang C., Zhang L., Guo Q., Zuo Y., Wang N., Wang H., Kong D., Zhu D., Zhang L. (2021). Robust Nanovaccine Based on Polydopamine-Coated Mesoporous Silica Nanoparticles for Effective Photothermal-Immunotherapy Against Melanoma. Adv. Funct. Mater..

[71] Chen C., Tang W., Jiang D., Yang G., Wang X., Zhou L., Zhang W., Wang P. (2019). Hyaluronic acid conjugated polydopamine functionalized mesoporous silica nanoparticles for synergistic targeted chemo-photothermal therapy. Nanoscale.

[72] Xue Y., Niu W., Wang M., Chen M., Guo Y., Lei B. (2020). Engineering a Biodegradable Multifunctional Antibacterial Bioactive Nanosystem for Enhancing Tumor Photothermo-Chemotherapy and Bone Regeneration. ACS Nano.

[73] Chu Y., Liu F., Qin L., Pan Q. (2016). Remote Manipulation of a Microdroplet in Water by Near-Infrared Laser. ACS Appl. Mater. Interfaces.

[74] Liu D., Ma L., Liu L., Wang L., Liu Y., Jia Q., Guo Q., Zhang G., Zhou J. (2016). Polydopamine-Encapsulated Fe_3_O_4_ with an Adsorbed HSP70 Inhibitor for Improved Photothermal Inactivation of Bacteria. ACS Appl. Mater. Interfaces.

[75] Zheng R., Wang S., Tian Y., Jiang X., Fu D., Shen S., Yang W. (2015). Polydopamine-Coated Magnetic Composite Particles with an Enhanced Photothermal Effect. ACS Appl. Mater. Interfaces.

[76] Lin L.-S., Cong Z.-X., Cao J.-B., Ke K.-M., Peng Q.-L., Gao J., Yang H.-H., Liu G., Chen X. (2014). Multifunctional Fe_3_O_4_@Polydopamine Core–Shell Nanocomposites for Intracellular mRNA Detection and Imaging-Guided Photothermal Therapy. ACS Nano.

[77] Yu S., Li G., Liu R., Ma D., Xue W. (2018). Dendritic Fe_3_O_4_@Poly(dopamine)@PAMAM Nanocomposite as Controllable NO-Releasing Material: A Synergistic Photothermal and NO Antibacterial Study. Adv. Funct. Mater..

[78] Choi C.K.K., Chiu Y.T.E., Zhuo X., Liu Y., Pak C.Y., Liu X., Tse Y.-L.S., Wang J., Choi C.H.J. (2019). Dopamine-Mediated Assembly of Citrate-Capped Plasmonic Nanoparticles into Stable Core–Shell Nanoworms for Intracellular Applications. ACS Nano.

[79] Wang S., Zhao X., Wang S., Qian J., He S. (2016). Biologically Inspired Polydopamine Capped Gold Nanorods for Drug Delivery and Light-Mediated Cancer Therapy. ACS Appl. Mater. Interfaces.

[80] Aguilar-Ferrer D., Vasileiadis T., Iatsunskyi I., Ziółek M., Żebrowska K., Ivashchenko O., Błaszkiewicz P., Grześkowiak B., Pazos R., Moya S. (2023). Understanding the Photothermal and Photocatalytic Mechanism of Polydopamine Coated Gold Nanorods. Adv. Funct. Mater..

[81] Nam J., Son S., Ochyl L.J., Kuai R., Schwendeman A., Moon J.J. (2018). Chemo-photothermal therapy combination elicits anti-tumor immunity against advanced metastatic cancer. Nat. Commun..

[82] Zhang L., Su H., Cai J., Cheng D., Ma Y., Zhang J., Zhou C., Liu S., Shi H., Zhang Y. (2016). A Multifunctional Platform for Tumor Angiogenesis-Targeted Chemo-Thermal Therapy Using Polydopamine-Coated Gold Nanorods. ACS Nano.

[83] Liu S., Wang L., Lin M., Wang D., Song Z., Li S., Ge R., Zhang X., Liu Y., Li Z. (2017). Cu (II)-Doped Polydopamine-Coated Gold Nanorods for Tumor Theranostics. ACS Appl. Mater. Interfaces.

[84] Han X., Xu Y., Li Y., Zhao X., Zhang Y., Min H., Qi Y., Anderson G.J., You L., Zhao Y. (2019). An Extendable Star-Like Nanoplatform for Functional and Anatomical Imaging-Guided Photothermal Oncotherapy. ACS Nano.

[85] Ma G., Zhang X., Zhao K., Zhang S., Ren K., Mu M., Wang C., Wang X., Liu H., Dong J. (2024). Polydopamine Nanostructure-Enhanced Water Interaction with pH-Responsive Manganese Sulfide Nanoclusters for Tumor Magnetic Resonance Contrast Enhancement and Synergistic Ferroptosis–Photothermal Therapy. ACS Nano.

[86] Tan X., Wang X., Zhang L., Liu L., Zheng G., Li H., Zhou F. (2019). Stable and Photothermally Efficient Antibody-Covered Cu_3_(PO4)_2_@Polydopamine Nanocomposites for Sensitive and Cost-Effective Immunoassays. Anal. Chem..

[87] Li Z., Hu Y., Howard K.A., Jiang T., Fan X., Miao Z., Sun Y., Besenbacher F., Yu M. (2016). Multifunctional Bismuth Selenide Nanocomposites for Antitumor Thermo-Chemotherapy and Imaging. ACS Nano.

[88] Yang J.-C., Chen Y., Li Y.-H., Yin X.-B. (2017). Magnetic Resonance Imaging-Guided Multi-Drug Chemotherapy and Photothermal Synergistic Therapy with pH and NIR-Stimulation Release. ACS Appl. Mater. Interfaces.

[89] Liu J., Jin L., Wang Y., Ding X., Zhang S., Song S., Wang D., Zhang H. (2018). A New Co-P Nanocomposite with Ultrahigh Relaxivity for In Vivo Magnetic Resonance Imaging-Guided Tumor Eradication by Chemo/Photothermal Synergistic Therapy. Small.

[90] Kong J., Ma S., Chu R., Liu J., Yu H., Mao M., Ge X., Sun Y., Wang Y. (2024). Photothermal and Photocatalytic Glycol Chitosan and Polydopamine-Grafted Oxygen Vacancy Bismuth Oxyiodide (BiO_1-x_I) Nanoparticles for the Diagnosis and Targeted Therapy of Diabetic Wounds. Adv. Mater..

[91] Guo W., Wang F., Ding D., Song C., Guo C., Liu S. (2017). TiO_2–x_ Based Nanoplatform for Bimodal Cancer Imaging and NIR-Triggered Chem/Photodynamic/Photothermal Combination Therapy. Chem. Mater..

[92] Ding X., Liu J., Li J., Wang F., Wang Y., Song S., Zhang H. (2016). Polydopamine coated manganese oxide nanoparticles with ultrahigh relaxivity as nanotheranostic agents for magnetic resonance imaging guided synergetic chemo-/photothermal therapy. Chem. Sci..

[93] Chu X., Zhang L., Li Y., He Y., Zhang Y., Du C. (2023). NIR Responsive Doxorubicin-Loaded Hollow Copper Ferrite @ Polydopamine for Synergistic Chemodynamic/Photothermal/Chemo-Therapy. Small.

[94] Yang Y., Liu J., Liang C., Feng L., Fu T., Dong Z., Chao Y., Li Y., Lu G., Chen M. (2016). Nanoscale Metal–Organic Particles with Rapid Clearance for Magnetic Resonance Imaging-Guided Photothermal Therapy. ACS Nano.

[95] Wu Q., Niu M., Chen X., Tan L., Fu C., Ren X., Ren J., Li L., Xu K., Zhong H. (2018). Biocompatible and biodegradable zeolitic imidazolate framework/polydopamine nanocarriers for dual stimulus triggered tumor thermo-chemotherapy. Biomaterials.

[96] Liu B., Li C., Xing B., Yang P., Lin J. (2016). Multifunctional UCNPs@PDA-ICG nanocomposites for upconversion imaging and combined photothermal/photodynamic therapy with enhanced antitumor efficacy. J. Mater. Chem. B.

[97] He H., Markoutsa E., Zhan Y., Zhang J., Xu P. (2017). Mussel-inspired PLGA/polydopamine core-shell nanoparticle for light induced cancer thermochemotherapy. Acta Biomater..

[98] Poinard B., Lam S.A.E., Neoh K.G., Kah J.C.Y. (2019). Mucopenetration and biocompatibility of polydopamine surfaces for delivery in an Ex Vivo porcine bladder. J. Control. Release.

[99] Ding Y., Su S., Zhang R., Shao L., Zhang Y., Wang B., Li Y., Chen L., Yu Q., Wu Y. (2017). Precision combination therapy for triple negative breast cancer via biomimetic polydopamine polymer core-shell nanostructures. Biomaterials.

[100] Zhang R., Su S., Hu K., Shao L., Deng X., Sheng W., Wu Y. (2015). Smart micelle@polydopamine core–shell nanoparticles for highly effective chemo–photothermal combination therapy. Nanoscale.

[101] Ding F., Gao X., Huang X., Ge H., Xie M., Qian J., Song J., Li Y., Zhu X., Zhang C. (2020). Polydopamine-coated nucleic acid nanogel for siRNA-mediated low-temperature photothermal therapy. Biomaterials.

[102] Gholami Derami H., Gupta P., Weng K.-C., Seth A., Gupta R., Silva J.R., Raman B., Singamaneni S. (2021). Reversible Photothermal Modulation of Electrical Activity of Excitable Cells using Polydopamine Nanoparticles. Adv. Mater..

[103] Zhao X., Zha X.-J., Tang L.-S., Pu J.-H., Ke K., Bao R.-Y., Liu Z.-y., Yang M.-B., Yang W. (2019). Self-assembled core-shell polydopamine@MXene with synergistic solar absorption capability for highly efficient solar-to-vapor generation. Nano Res..

[104] Fan R., Chen C., Hou H., Chuan D., Mu M., Liu Z., Liang R., Guo G., Xu J. (2021). Tumor Acidity and Near-Infrared Light Responsive Dual Drug Delivery Polydopamine-Based Nanoparticles for Chemo-Photothermal Therapy. Adv. Funct. Mater..

[105] Liu S., Pan J., Liu J., Ma Y., Qiu F., Mei L., Zeng X., Pan G. (2018). Dynamically PEGylated and Borate-Coordination-Polymer-Coated Polydopamine Nanoparticles for Synergetic Tumor-Targeted, Chemo-Photothermal Combination Therapy. Small.

[106] Yuan Z., Lin C., He Y., Tao B., Chen M., Zhang J., Liu P., Cai K. (2020). Near Infrared Light Triggered Nitric Oxide-Enhanced Photodynamic Therapy and Low-Temperature Photothermal Therapy for Biofilm Elimination. ACS Nano.

[107] Wang Z., Wang L., Prabhakar N., Xing Y., Rosenholm J.M., Zhang J., Cai K. (2019). CaP coated mesoporous polydopamine nanoparticles with responsive membrane permeation ability for combined photothermal and siRNA therapy. Acta Biomater..

[108] Zeng W., Li Z., Huang Q., Ding C., Yang L., Wang W., Shi Z., Yang Y., Chen H., Mei L. (2024). Multifunctional Mesoporous Polydopamine-Based Systematic Delivery of STING Agonist for Enhanced Synergistic Photothermal-Immunotherapy. Adv. Funct. Mater..

[109] Liu S., Liu Y., Chang Q., Celia C., Deng X., Xie Y. (2024). pH-Responsive Sorafenib/Iron-Co-Loaded Mesoporous Polydopamine Nanoparticles for Synergistic Ferroptosis and Photothermal Therapy. Biomacromolecules.

[110] Chen W., Song Y., Bai S., He C., Guo Z., Zhu Y., Zhang Z., Sun X. (2023). Cloaking Mesoporous Polydopamine with Bacterial Membrane Vesicles to Amplify Local and Systemic Antitumor Immunity. ACS Nano.

[111] Ge R., Lin M., Li X., Liu S., Wang W., Li S., Zhang X., Liu Y., Liu L., Shi F. (2017). Cu^2+^-Loaded Polydopamine Nanoparticles for Magnetic Resonance Imaging-Guided pH- and Near-Infrared-Light-Stimulated Thermochemotherapy. ACS Appl. Mater. Interfaces.

[112] Miao Z.-H., Wang H., Yang H., Li Z.-L., Zhen L., Xu C.-Y. (2015). Intrinsically Mn^2+^-Chelated Polydopamine Nanoparticles for Simultaneous Magnetic Resonance Imaging and Photothermal Ablation of Cancer Cells. ACS Appl. Mater. Interfaces.

[113] Zou Y., Wu T., Li N., Guo X., Li Y. (2020). Photothermal-enhanced synthetic melanin inks for near-infrared imaging. Polymer.

[114] Xue C., Li M., Liu C., Li Y., Fei Y., Hu Y., Cai K., Zhao Y., Luo Z. (2021). NIR-Actuated Remote Activation of Ferroptosis in Target Tumor Cells through a Photothermally Responsive Iron-Chelated Biopolymer Nanoplatform. Angew. Chem. Int. Ed..

[115] Wang Z., Wang W., Wamsley M., Zhang D., Wang H. (2022). Colloidal Polydopamine Beads: A Photothermally Active Support for Noble Metal Nanocatalysts. ACS Appl. Mater. Interfaces.

[116] Xu X., Sarhan R.M., Mei S., Kochovski Z., Koopman W., Priestley R.D., Lu Y. (2023). Photothermally Triggered Nanoreactors with a Tunable Catalyst Location and Catalytic Activity. ACS Appl. Mater. Interfaces.

[117] Wang X., Zhang J., Li H., Zhang R., Yang X., Li W., Li Z., Gu Z., Li Y. (2024). Quaternary Ammonium Assisted Synthesis of Melanin-like Poly(l-DOPA) Nanoparticles with a Boosted Photothermal Effect. ACS Appl. Mater. Interfaces.

[118] Bai W., Yang P., Zhang H., Wang T., Yang Y., Zhang X., Duan G., Xu Y., Li Y. (2023). Polycondensation-Involved Melanin-like Polymers for Enhanced Solar Energy Utilization. Macromolecules.

[119] Han L., Zhang Y., Lu X., Wang K., Wang Z., Zhang H. (2016). Polydopamine Nanoparticles Modulating Stimuli-Responsive PNIPAM Hydrogels with Cell/Tissue Adhesiveness. ACS Appl. Mater. Interfaces.

[120] Gao G., Jiang Y.-W., Jia H.-R., Wu F.-G. (2019). Near-infrared light-controllable on-demand antibiotics release using thermo-sensitive hydrogel-based drug reservoir for combating bacterial infection. Biomaterials.

[121] Luo Y., Wei X., Wan Y., Lin X., Wang Z., Huang P. (2019). 3D printing of hydrogel scaffolds for future application in photothermal therapy of breast cancer and tissue repair. Acta Biomater..

[122] Wu Y., Wang H., Gao F., Xu Z., Dai F., Liu W. (2018). An Injectable Supramolecular Polymer Nanocomposite Hydrogel for Prevention of Breast Cancer Recurrence with Theranostic and Mammoplastic Functions. Adv. Funct. Mater..

[123] Zhao X., Liang Y., Huang Y., He J., Han Y., Guo B. (2020). Physical Double-Network Hydrogel Adhesives with Rapid Shape Adaptability, Fast Self-Healing, Antioxidant and NIR/pH Stimulus-Responsiveness for Multidrug-Resistant Bacterial Infection and Removable Wound Dressing. Adv. Funct. Mater..

[124] Yang P., Bai W., Zou Y., Zhang X., Yang Y., Duan G., Wu J., Xu Y., Li Y. (2023). A melanin-inspired robust aerogel for multifunctional water remediation. Mater. Horiz..

[125] Wu X., Cao S., Ghim D., Jiang Q., Singamaneni S., Jun Y.-S. (2021). A thermally engineered polydopamine and bacterial nanocellulose bilayer membrane for photothermal membrane distillation with bactericidal capability. Nano Energy.

[126] Zhang C., Wu M.-B., Wu B.-H., Yang J., Xu Z.-K. (2018). Solar-driven self-heating sponges for highly efficient crude oil spill remediation. J. Mater. Chem. A.

[127] Zhou Z., Wang X., Yu H., Yu C., Zhang F. (2022). Dynamic Cross-Linked Polyurea/Polydopamine Nanocomposites for Photoresponsive Self-Healing and Photoactuation. Macromolecules.

[128] Li Z., Yang Y., Wang Z., Zhang X., Chen Q., Qian X., Liu N., Wei Y., Ji Y. (2017). Polydopamine nanoparticles doped in liquid crystal elastomers for producing dynamic 3D structures. J. Mater. Chem. A.

[129] Wu J., Xu Y., Wu D., Zhou W., Wang P., Gong J., Yang J., Xia X. (2025). Melanin/melanin-like nanoparticles in tumor photothermal and targeted therapies. Int. J. Pharm..

[130] Menichetti A., Vicenzi S., Pane A., Mordini D., Mancin F., Montalti M. (2025). Photothermal Release by Melanin-like Nanoparticles: Biomedical Applications. J. Funct. Biomater..

[131] Guzman-Sanchez S., Patel N., Rosado A.S. (2025). Recent progress of polydopamine nanoparticles as advanced antimicrobial nanomaterials. Front. Bioeng. Biotechnol..

[132] Ma Z.-Y., Li D.-Y., Jia X., Wang R.-L., Zhu M.-F. (2023). Recent Advances in Bio-Inspired Versatile Polydopamine Platforms for “Smart” Cancer Photothermal Therapy. Chin. J. Polym. Sci..

[133] Li M., Sun X., Zhang N., Wang W., Yang Y., Jia H., Liu W. (2018). NIR-Activated Polydopamine-Coated Carrier-Free “Nanobomb” for In Situ On-Demand Drug Release. Adv. Sci..

[134] Wang Y., Wei G., Zhang X., Huang X., Zhao J., Guo X., Zhou S. (2018). Multistage Targeting Strategy Using Magnetic Composite Nanoparticles for Synergism of Photothermal Therapy and Chemotherapy. Small.

[135] Qi X., Huang Y., You S., Xiang Y., Cai E., Mao R., Pan W., Tong X., Dong W., Ye F. (2022). Engineering Robust Ag-Decorated Polydopamine Nano-Photothermal Platforms to Combat Bacterial Infection and Prompt Wound Healing. Adv. Sci..

[136] Yuan G., Cen J., Liao J., Huang Y., Jie L. (2021). In situ hydrogen nanogenerator for bimodal imaging guided synergistic photothermal/hydrogen therapies. Nanoscale.

[137] Zhang C., Zheng D.-W., Li C.-X., Zou M.-Z., Yu W.-Y., Liu M.-D., Peng S.-Y., Zhong Z.-L., Zhang X.-Z. (2019). Hydrogen gas improves photothermal therapy of tumor and restrains the relapse of distant dormant tumor. Biomaterials.

[138] Wang W., Li M., Huang X., Fang J., Peng F., Huang H. (2022). Structural evolution mechanisms of Polydopamine/CdS and photothermal effect boosted photocatalytic H_2_ production activity. Appl. Surf. Sci..

[139] Lin Q., Yang Y., Ma Y., Zhang R., Wang J., Chen X., Shao Z. (2018). Bandgap Engineered Polypyrrole–Polydopamine Hybrid with Intrinsic Raman and Photoacoustic Imaging Contrasts. Nano Lett..

[140] Liang R., Yu H., Wang L., Wang N., Amin B.U. (2021). NIR Light-Triggered Shape Memory Polymers Based on Mussel-Inspired Iron–Catechol Complexes. Adv. Funct. Mater..

[141] Xie W., Dhinojwala A., Gianneschi N.C., Shawkey M.D. (2024). Interactions of Melanin with Electromagnetic Radiation: From Fundamentals to Applications. Chem. Rev..

[142] Wang C., Zhang R., Zhang J., Zhang X., Zhang H., Cao H., Yang Z., Li Y. (2024). Synthetic fungal melanin UV absorbers. Sci. China Chem..

[143] Hong S., Kim J., Na Y.S., Park J., Kim S., Singha K., Im G.-I., Han D.-K., Kim W.J., Lee H. (2013). Poly (norepinephrine): Ultrasmooth material-independent surface chemistry and nanodepot for nitric oxide. Angew. Chem. Int. Ed..

[144] D’Alba L., Van Hemert C., Spencer K.A., Heidinger B.J., Gill L., Evans N.P., Monaghan P., Handel C.M., Shawkey M.D. (2014). Melanin-Based Color of Plumage: Role of Condition and of Feathers’ Microstructure. Integr. Comp. Biol..

[145] Shawkey M.D., D’Alba L., Xiao M., Schutte M., Buchholz R. (2015). Ontogeny of an iridescent nanostructure composed of hollow melanosomes. J. Morphol..

